# Bioethics, Sociality, and Mental Illness

**DOI:** 10.1093/jmp/jhad002

**Published:** 2023-04-20

**Authors:** Magnus Englander

**Affiliations:** Malmö University, Malmö, Sweden

**Keywords:** empathy, mental illness, phenomenology, qualitative research

## Abstract

The phenomenology of bioethics is approached here in relation to the lived experience as it relates to the everyday lifeworld of persons suffering from mental illness. Taking a road less traveled, the purpose here is to elucidate ethical issues relating to sociality, using findings from qualitative phenomenological psychological research. Qualitative studies of schizophrenia and postpartum depression serve as examples. Layered throughout is the applied phenomenological argument pointing to the importance of returning to mundane intersubjectivity and the reversibility between mental illness, the existential context of suffering, and sociality.

“…there is no inner man, man is in the world, and only in the world does he know himself.”

Maurice Merleau-Ponty, Preface, *Phenomenology of Perception*, 1962

## I . INTRODUCTION

What constitutes phenomenological bioethics? Svenaeus has recently provided us with the following account:

Phenomenological bioethics can be regarded as the part of the phenomenology of medicine and health care that focuses on ethical dilemmas arising in connection with understanding and helping suffering persons, and in connection with dealing with medical-technological dilemmas involving human bodies and their parts. (2018, 6)

I will go along with Svenaeus’s first sense of phenomenological bioethics, although I will not be concerned specifically with ethical dilemmas. Instead, I will approach bioethics as a type of reflection that has something in common with the phenomenological stance, that is, that we can never take for granted that the natural sciences and modern medicine are anything else than a human activity. In the following, will be concerned with ethical reflection on our moral responsibility of sociality and how it relates to the existential context of mental illness. To clarify, there is no attempt here to dispute the effort of the natural sciences and the quest to at one point in time discover the cause of mental illness. Already in *Ideas Pertaining to a Pure Phenomenology and to Phenomenological Philosophy*, Edmund Husserl wrote, “When it is actually natural science that speaks, we listen gladly and as disciples” (1998, 39). The point here is to guard against taking something for granted, and therefore risk the loss of openness and the reflection of empathy toward the existential situation of the suffering other. The overall question considered is: In what specific way could applied phenomenological inquiry make a contribution to ethical reflection within the world of modern psychiatry? The most common way to approach that question would be for the applied phenomenologist to look for ethical analyses in the works of Husserl, Heidegger, Merleau-Ponty, Sartre, and others and apply these insights to dilemmas and concerns found within, for example, modern psychological and psychiatric practice and research, as well as to related technological advancements regarding treatment and care. However, I will follow a road less traveled. By exemplifying using research findings from qualitative phenomenological psychological studies in psychopathology, I shall seek to clarify how ethical issues are already intertwined in the sociality of the everyday lifeworld.

## II. QUALITATIVE PHENOMENOLOGICAL RESEARCH AND PSYCHOPATHOLOGY

Historically, qualitative research springs from the anthropological method known as ethnography, seeking the discovery of social constitution. The original purpose of ethnography, as following the method of participant observation^1^, is the essence of qualitative research in the psychological and social sciences; even though some qualitative methods have deviated from this purpose and have self-destructively turned its inquiry into the verification of postmodern theories or made itself into some version of a survey study without statistics.^2^ The problems surrounding participant observation have been debated since its association with Malinowski’s (1922) study of the Kula. Nevertheless, the problem of knowing others is only a problem in so far as one is posing such a question from the stance of naturalism.^3^ To naturalize intentionality in its relation to intersubjectivity means that knowing others turns into a problem, in which we become non-related entities and what Husserl would have referred to as “windowless monads” (see Staiti, 2014). Perhaps we could call this “the hard problem of empathy,” simply because it mimics the “the hard problem of consciousness,” as famously raised by Chalmers (1995). From a phenomenological perspective, and as explicated by Varela (1996), the “hard problem of consciousness” is only a problem because the question is posed from the stance of naturalism. Varela’s (1996) well-known remedy for the “hard problem” turned into the research program of neurophenomenology, focusing on accessing lived experience using a phenomenological methodology. According to Bitbol (2021), neurophenomenology calls for an “existential attitude”^4^ beginning from Merleau-Ponty’s ontology of *reversibility*.^5^ Such a point of departure is congruent with an ethnographic phenomenology in which knowing others is not a problem related to empirical criteria, as if the meaning resided *inside* the space of the body or psyche of the other in the same sense that a table or chair is in a room. As Heidegger once wrote “the problem of empathy is just as absurd as the question of the reality of the external world” (1985, 240). But not to forget, and as voiced by Husserl (1999), this is not to say that anyone could have the actual experience of another person as in *being* the other, but instead, as Giorgi has consistently pointed out in relation to qualitative phenomenological research, “the object always transcends the act in which it appears” (1997, 237). It is hardly a surprise then that some research traditions have tried to merge the original purpose of ethnography and gone further into the constitution of the everyday lifeworld by turning specifically to insights and methods within the phenomenological tradition (e.g., Giorgi, 1985; Aschworth, 1995; Davidson, 2003; Churchill, 2022). For example, Husserl’s (1970) original dictum “to go back to the matters themselves” was contextualized within qualitative inquiry as participant observation by Giorgi, as he stated, “for a phenomenological psychologist one interpretation of that expression means to go to the everyday world where people are living through various phenomena in actual situations” (1985, 8). Following such a trajectory, that is, by turning to the everyday lifeworld and to person’s suffering from mental illness, Larry Davidson has consistently argued for a phenomenological approach to a qualitative inquiry as a way to remind the world of psychiatry that it cannot separate itself from sociality. Davidson writes,

… humans develop a sense of self *in relation to* others. I first come to know myself as a person separate from others *through* my relationships with them, and come to form a sense of identity that is heavily influenced by how those others view and treat me. There can be no “I” without at least one “you” that together constitute a “we”. This interdependence of the social and personal—what has come to be considered a fundamental fact of infant development discovered through close observation, and taken as the basis for hypotheses about the neural origins of identity—was argued on a philosophical basis by Husserl nearly a century ago. (2018, 9)

Thus, Davidson makes the point that the ego is already intertwined in a community. In other words, we would not be able to understand that somebody was different from ourselves if we were not already operating on the intersubjective ground of intentionality.

Psychopathological phenomena can thus never be fully understood as an individual problem, because a person is always somehow intersubjectively related to a social world. Of course, this is not just the case in relation to mental illness, but for any type of person suffering from any type of illness, disease, or malady. However, mental illness has a peculiar type of stigma surrounding it. Take, for example, hallucinations and delusions as part of the diagnosis of schizophrenia, in which a sense of agency or even ownership in relation to one’s experiential sense of self is lost (see Gallagher, 2005). The ambiguity of such a condition clearly affects social relations different from, let us say, the loss of agency when one has a broken leg (although not to imply that malady lacks connection to social stigma). The psychosocial world constitutes a type of reversibility between others and the person suffering from mental illness that also contributes to the constitution of the illness. This was recognized by Karl Jaspers already in 1913, who wrote,

The transmission of culture like the entire life of man is accomplished within a community. The individual reaches his fulfillment and finds his place, meaning, and field of activity in the community in which he lives. The tensions between himself and the community are one of the understandable sources of his psychiatric disturbances. Every moment of the day the community is effectively present for every individual. (1997, 710)

The *tension* that Jaspers describes and the experience that the *community is always present* for the person suffering from mental illness becomes key in understanding how the everyday lifeworld and mental illness are existentially constituted as a reversibility between the lived experience and the social world.

Certainly, there are several aspects of reversibility that could be addressed in relation to qualitative research into mental illness; however, one particular point seems to stand out, which is the issue of how the ethnographic purpose driving social research relates to phenomenological philosophy. Merleau-Ponty writes,

Philosophy is nature in us, the others in us, and we in them. Accordingly, we must not simply say that philosophy is compatible with sociology, but that it is necessary to it as a constant reminder of its tasks; and that each time the sociologist returns to the living sources of his knowledge, to what operates within him as a means of understanding the forms of culture most remote from him, he practices philosophy spontaneously. Philosophy is not a particular body of knowledge; it is the vigilance which does not let us forget the source of all knowledge. (1964, 110)^6^

In other words, neither the qualitative social researcher, nor the philosopher, operate in a closed-off space, detached from either philosophy or science, but are in constant reversibility of such. Perhaps we can suggest that the world of psychiatry is already with us intersubjectively within our community, meaning that ethical issues in relation to sociality are already present and something that we can directly philosophically reflect upon. Even when seeking a sense of recovery from serious mental illness, there is no escape from the reversibility between the lived experience and our sociality. As Davidson writes,

[The] foundation for recovery is not something a person can provide for himself or herself. I cannot simply will myself to have a sense of belonging, I cannot make other people accept me within their ranks, I cannot establish a foundation for belonging for myself by myself. Having a sense of belonging necessarily requires the caring, compassionate actions of others, it requires others to treat me like “a human” (as opposed to “an unhuman”), it requires being included in the life of one’s community as a valued and contributing member rather than being shunned, ignored, or relegated to the margins of society. (2018, 20)

As should be evident by now, seeing qualitative research from the grounds of a phenomenological theory of science and an ethnographic purpose, we are not talking about qualitative research as in the attempt at verifying a postmodern theory or conceptually sorting themes into some sort of content analysis, but experientially and with a focus on meaning constitution turning to and disclosing the reciprocal constitution between the other’s lived experience and the existential context of the social world.

The relationship between qualitative research and phenomenological philosophy has been debated for over a half a decade and has intensified lately between human scientists and philosophers (for a more extensive coverage of this recent debate, see Morley, 2019; Zahavi, 2019, 2021; Zahavi and Martiny, 2019; Englander, 2020;  Giorgi, 2020, 2021). Even though this is not the place to go further into such methodological matters, such debates are often grounded in problems relating to the basic relationship between philosophy and social science. As stated earlier, discovery-oriented, qualitative research has its roots in the ethnographic purpose whereas phenomenology has its roots in the solitary, reflective work of the philosopher. Combining the two is no easy task, but there is some common ground and that is its motivation to “go to the matters themselves” and to search for the intersubjective constitution, even though the social sciences remain within mundane intersubjectivity (Schutz, 1962, 1967), perhaps only visiting the transcendental realm when necessary (see Natanson, 1973). And even Husserl seemed to have been well aware of the interdependency between philosophy and social science. As pointed out by Merleau-Ponty,

…in the complete works of Husserl, the letter that he wrote to Lévy-Bruhl on March 11, 1935, after having read *La mythologie primitive*. Here he seems to admit that the philosopher could not possibly have immediate access to the universal by reflection alone—that he is in no position to do without anthropological experience or to construct what constitutes the meaning of other experiences and civilizations by a purely imaginary variation of his own experiences. “It is a possible and highly important task,” he writes, “it is a great task to project ourselves into (*einzufühlen*) a human community enclosed in its living and traditional sociality, and to understand it insofar as, in and on the basis of its total social life, that human community possesses the world which is not for it a ‘representation of the world’ but the real world.” (1964, 107–8)

The relationship between philosophy and social science is a complex one, however, it seems necessary to at least acknowledge the inevitable reversibility between what presents itself within the social context and the lived experience of phenomena. As such, it does not come as a surprise that qualitative phenomenological research could contribute to the study of ethics as it relates to mental illness. In an article by Wertz (2011), there is a direct proposal that the social world and qualitative research are related to ethics. Qualitative research in the postmodern era has been driven by an emancipatory aim, providing us with findings about phenomena based upon the lived experience of the people who have been the ones who have been suffering from all kinds of conditions, all within the context of a community and a society. On the negative side, qualitative research has at times lost track of a critical epistemology and become part of a postmodern era of relativism, making certain qualitative methods ignore the systematization of a theory of science and the phenomenological battle against a representational theory of mind (Applebaum, 2012; Englander, 2019). However, one can also probe deeper into the value of the qualitative research movement. As Wertz writes,

As moral values are reinstated, science appears to become subordinated to political power … however, … what may cynically appear to be reducible to shear political power play and ideology, when cast in the light of an ethically grounded epistemology, may be better understood as an obligation required by authentic human scientific knowledge. (2011, 84)

In other words, ethics does not have to equal politics or be subordinated to epistemology (and only added as ethical guidelines), but instead be understood as a type of phenomenological reflection of embodied, lived experience and its reversibility to sociality.

## III. QUALITATIVE PHENOMENOLOGICAL RESEARCH FINDINGS AND ETHICAL REFLECTION

How can we then see how ethical issues are already present in qualitative research in psychiatry? The following example is an excerpt from Davidson’s (2003) book *Living Outside Mental Illness*. A person with schizophrenia is interviewed about the phenomenon, “to live with schizophrenia.” However, Davidson’s study is concerned with the phenomenon, *recovery from mental illness*, that is, in how one goes from “inside the world of schizophrenia” to “living outside the world of schizophrenia,” meaning that our example only covers one aspect of the overall phenomenon being studied. The whole methodology and analysis cannot be accounted for here; nevertheless, we have selected one constituent (or “essential part”) of the meaning structure of the phenomenon and traced it back to a moment of one of the participants being interviewed. Davidson reports,

… when asked how she felt she was treated in the community, replied: “People treat me like not a person.” When asked “How’s that?” by an interviewer who was looking for clarification and details, she replied: “They ignore me … [they treat me] like crap. I felt like I belonged [in school], but now I don’t feel I belong anywhere,” (2003, 154)

By taking this fragment of the interview, we are able to use it as an example here and thus to clarify its connection to the qualitative findings, that is, after Davidson has elucidated the phenomenon based on all his ethnographic interview material. Davidson provides the following reflection on his discovery of one of the constituents of the phenomenon described as “not being treated as a person and not feeling as one belongs anywhere.” He writes,

… this aspect of schizophrenia is as difficult to get a handle on as the loss of agency …. That is, we have found this experience to occur at the same kind of foundational level as the person’s loss of self; a basic sense of loss that most of us will not have experienced first hand. (Davidson, 2003, 154)

As we can see, the first-person focus on the loss of self and a sense of agency (often reported in phenomenological accounts of schizophrenia) can now also be disclosed in how such experiences are socially intertwined. Often, as phenomenological psychiatrists have pointed out, the psychiatric condition known as schizophrenia often comes with experiences in which this basic sense of subject-hood is not experienced. For example, Henriksen reports on a patient’s experience as “the body feels awkward as if it does not really fit. It feels like the body is not really me, as if it is rather a machine controlled by my brain, as if the body is a mere appendage” (2018, 38). The reversibility between such typical experiences of schizophrenia and the way that these patients are situated in the community and also how they are generally approached by a medical community needs to be reflected upon. As Fuchs states,

The neurobiologically informed concept of human beings affects the life-world and changes our self-understanding in everyday life. As a result of a gradual process of self-reification we start to see ourselves less as human beings taking decisions based on reason or motives, but rather as agents of our genes, hormones, and neurons. (2018, xiii)

What is lost in such a description is not subjectivity as such, because that would simply be impossible, but it is the value that we give to the volitional body. To then also take into consideration the qualitative finding, “not being treated as a person and not feeling as one belongs anywhere” and how this social meaning relates to a loss of the volition of the person raises ethical concerns and the complexity of all layers of reversibility related to suffering from schizophrenia in everyday life. Qualitative studies also awaken the ethical possibility of transforming the psychiatric context with its pre-conceived medical focus of the body as a thing to a human encounter, including the reversibility of the lived body with a sociality. Ferrarello writes,

Becoming alive means seeing our own alienation and engaging this alien [body as a thing] in a dialogue. How are we treating our body? What part of it is in pain? Where does the place of our pleasure reside? Are we breathing? It is from this encounter that we become living things; we awaken from a condition of thing-hood to one of subject-hood. (2018, 168)

In such a sense, qualitative phenomenological inquiry is not disconnected from ethics, but awakens a reflection on personhood, which includes a reflection on the reversibility between person and community.

Davidson’s (2003) study can help us to understand that feelings of alienation toward others is not just encapsulated in some schizophrenic solipsism and caused by biological events, but also unfolds within a social context. In other words, the lived experience of alienation also relates to the psychological meaning of others in an interpersonal, social world. Davidson elaborates on the social, ethical aspects in the following way:

It is very possible … that people with schizophrenia may find the inevitable peer group of other people with serious mental illness as the most accepting social sphere to which they have access, and, with that, the “mental patient” identity that comes along with this affiliation. In many cases, it will be preferable to come to see oneself as a mental patient who can socialize with other mental patients than to see oneself as a nobody who belongs nowhere. As one elderly participant described the limited kind of social interaction he had during a long-term inpatient stay that seemed to follow him out into the community following discharge: “I had nothing in common with the other patients except that I was another nut with them, you know.” If you feel that you don’t belong to the broader world … then it is understandable how you would come to limit your social interactions to hanging out with other people who share your tragic fate. (2003, 154–5)

Thus, the phenomenon of living with schizophrenia is not just a neurobiological or an individual psychopathological phenomenon, but also a phenomenon situated in the existential context of the social world and concretely in the community in which we all live. In the social world where psychological and psychopathological phenomena appear, mundane intersubjectivity becomes its necessary background in order to understand how schizophrenia relates to our sense of normativity and sociality.

Another example of findings from a qualitative phenomenological study that addresses ethical reflection in the world of psychiatry is the extensive research on postpartum depression conducted by Idun Røseth (2013). Even here we see the attempt at naturalizing the condition, so that the reversibility of the person with her community becomes secondary. In a recent article, Røseth and Boongaardt (2018) summarize the typical naturalistic attitude in which the mother is fitted into DSM-criteria^7^ as depressed and hypothesized to suffer from hormonal dysregulation because of genetic susceptibility. Røseth and Bongaardt conclude “In the dominant psychiatric tradition, postpartum depression is primarily treated as something inherent in the individual, but at the same time something separate from the person” (2018, 121). This is not to say that neither postpartum depression nor schizophrenia should not be treated as a medical condition and that a prospective cure is not plausible, but to elucidate the taken-for-grantedness of the medical model as the ground in how to approach ethical issues in relation to such human conditions. Beginning instead with the reversibility between the lived experience and the world, we would be able to include the medical model as a technical tool toward help. However, such a stance would also allow us to remain within the concrete and practical world, in which we would be able to co-participate with the other’s lived meaning, thus also aiding our ethical reflection, often long before any diagnosis has been set and any treatment has been administered. To illustrate this point further, let us take a look at some of the findings in Røseth’s (2013) qualitative phenomenological study on postpartum depression. Røseth, Binder, and Malt write,

Crumbling under a world perceived as dangerous, the mother fears for her baby’s safety. She is tormented by a painful feeling of insecurity and anxiety that infiltrates normal everyday activities. Her anxiety is nourished by her own emotional past and present; however, she is mostly unaware of this process. The lived body manifests itself as an obstacle by its heaviness and lack of energy. She feels out of touch with the world, the baby, and other people. Painfully aware of this alienation, she tries to convince herself rationally of her love for the baby. Being unable to live up to her own expectations regarding love and care for her baby makes her vulnerable to the gaze of others. She feels guilt because she perceives her mothering skills as inadequate, and she feels shame because she perceives herself as inferior or bad. She transforms her experience of time into guilt for missed opportunities for loving and caring for her baby. She also grieves her baby’s loss of a loving caring mother. Anxiety or strong feelings of guilt and shame make the woman conceal her true thoughts and feelings and withdraw from social others. Although desperately in need of help to care for the baby, she often interprets support as a confirmation of her failure as a mother. In constraining herself by isolation she feels ambivalent. She fears social situations, but at the same time she feels terribly lonely, and thus longs for good social relations which she hopes can relieve her pain. (2011, 179–80)

As we can clearly see within this phenomenological psychological description, the reversibility between sociality and the lived experience clearly presents a moral dilemma for the mother.

Similar to the case of schizophrenia presented earlier, there seems to be a tendency to withdraw from any social interaction, which in terms of postpartum depression becomes a way out of other’s moral judgment. Røseth and Bongaardt elaborates on how the dilemma unfolds, as they write,

But what happens when a person becomes unable to connect in a habitual and synchronized manner with others? The structure of depression postpartum…reveals a fundamental change in how the mother exists in the world with others. The process of integrating and habituating *being with* the baby is not successfully resolved…The mother feels unable to fully integrate the vulnerable baby in herself; on the contrary, it presents as an obstacle to gain access to the world. She resolves this unfulfilled integration by caring for the baby in a “mechanical” way. She follows routines and does what must be done, solving problems analytically. But she experiences this as difficult and stressful; the world has lost its inviting pull and it does not afford engagement in social-cultural settings. (2018, 127)

The loss of the “inviting pull” is the loss of the existential reversibility that constitutes the interaction between the volitional body and the community. To use a rather harsh analogy, the meaning of the movement away from a community is similar to the movement of an injured animal pulling away from its herd, because it is about to die. In what way are we who are not suffering from mental illness morally responsible for doing something about this state of despair?

In one of Davidson’s (2003) qualitative studies there is one particular part of the findings that stands out and that can help to answer our previous question, perhaps providing us with ethical guidance. As we have indicated earlier, it should be noted that Davidson’s (2003) research is situated within the movement of a recovery-oriented psychiatry, in which recovery does not refer to “medical recovery,” but follows Anthony’s (1993) definition of recovery as a way to have the right to live a meaningful life within one’s community, even though symptoms of mental illness still prevail. Nevertheless, Davidson’s (2003) research shows that there is a shift or transition from the phenomenon of “living inside mental illness” to “living outside mental illness.” The essence of “living outside of mental illness” all points to the person being *socially included*. However, social inclusion cannot just be provided for by an abstract model, such as in establishing a place in society called psychiatric services or that one of the consequences of taking the medication (often targeting symptom relief) should automatically lead to meaningful social interaction. That is not to say that psychiatric services or medication are not helpful strategies in the path toward recovery. In fact, Davidson clearly indicates that medication contributes to the recovery process, as he states, “This fact is also well reflected in our interviews, with participants saying things like: ‘Without the medicine I wouldn’t be here right now. I would have committed suicide’” (2003, 159–60). In other words, even though the advancement of biotechnology is an essential part of the recovery process, the openness of the community constitutes the intersubjective ground in which it is made possible. Perhaps there is some truth to what the phenomenological psychiatrist Erwin Straus once wrote: “While the activities of the physician are directed generally to [hu]man as a living creature, to the organism and its function, the psychiatrist is concerned with man as a citizen of the historical and social world or worlds” (1969, 2).

## IV. CONCLUSIONS

It has been my purpose here to disclose how our reflection related to bioethics, in the sense of the reversibility between mental illness and sociality, could begin by approaching findings within qualitative phenomenological research. In such research, the ethnographic and applied phenomenological purpose meet on the grounds of mundane intersubjectivity, helping us to see how sociality, ethical issues, and psychological meanings are reversible constituted in the existential context of the everyday lifeworld. The two examples of schizophrenia and postpartum depression presented here illustrated ethical issues as something more existential about mental illness and sociality, that is, as movement-away-from-others, that is, as withdrawal of our fundamental situation of being-with-others and perhaps as a step toward a withdrawal of being-in-the-world. Our moral responsibility for these human beings is not just to be found in providing them with treatment, but as to see that our reflection seeks to elucidate what we take for granted in terms of social inclusion. Perhaps then we will discover that we are already in reversibility-with-others.

## Footnotes

1 Often, the sense of participant observation in anthropology is that it only applies to ethnographic field work. For a recent rebuttal of such a view in relation to psychological interviews, see Englander (2020). In short, participant observation might very well equal field work in sociological and anthropological qualitative research, however, in the world of psychology it relates to interviews in which there is a sense of participation in the personal world of the other. In ethnography, as used in anthropology, there is also a tendency to equal participant observation with having practical experience of the practical acts or “doings” of another culture or social world. As is common in the world of psychology, psychologists have often been extensively trained as participants in interviews or been through their own psychotherapy, therefore having experience in the psychological acts or “doings,” such as “what it is like” for the participant in the interview, that is, in which the personal world is being explored. For a more developed account on qualitative research interviews in the context of psychiatry, see Henriksen, Englander, and Nordgaard (2022).

2 For a recent critical take on general knowledge claims in relation to the wide variety of qualitative methods, see Englander (2019). See also, Giorgi (1970) and Applebaum (2012) on what constitutes a human scientific approach toward qualitative phenomenological psychological research.

3 To clarify, in Husserlian phenomenology, *naturalism* has been described by Drummond as: “The view that considers everything exclusively as a natural being, that is, as a spatio-temporal being enmeshed in a causally mechanistic, purely physical world. Whatever exists, on this view, exists as either a physical individual subject to causal laws or as a merely dependent function of physical variations, which variations are themselves governed by fixed laws. What is most problematic about naturalism in Husserl’s view is its tendency to reduce the psychic to the physical, thereby naturalizing consciousness and collapsing into psychologism. Naturalism, in other words, both fails to grasp the psychic properly and collapses all ideal laws, most importantly, those of logic, and all ideal norms into merely empirical laws and normative generalizations” (2010, 142).

4 By being open to that the patient is already situated in the social world we discover, according to Ratcliffe (2005), that there are existential feelings already present, and as part of the lived experience of the patient. By turning to the existential dimension of psychopathology enables us then to see what Aho (2019) has identified as the *context of suffering*. Consequently, and as Svenaeus has recently concluded (in his account of the phenomenology of bioethics): mental illness “… cannot be separated from cultural norms and existential quests …” (2018, 96).

5 Chiasm and the intertwining are terms that point to *reversibility* in Merleau-Ponty’s (1968) ontology. According to Dillon (1997, 155), “The figure called forth by these terms is that of the crossing and turning back on itself of the single thread that emanates from the spider’s body when she spins her web. This web-matrix, the whole cloth, the flesh, of the world is an interweaving, an elementary knotting, which is always prior to its unravelling in language and thought. The world is primordially phenomenon, primordially woven and weaving: and autochthonous organization, a Gestalt-contexture.” Applied to a phenomenological social psychiatry and qualitative phenomenological research, reversibility marks the intersubjective relation constituting a phenomenon as appearing for an embodied, lived experience within context of the social world (see Englander, 2018, 2019, 2020). To see how Merleau-Ponty’s basic notion of reversibility is understood as applied to neurophenomenology, see Bitbol (2021).

6 Jason Throop (2018) has recently presented this view of Merleau-Ponty as the possibility for “the ethnographic *epoché*.”

7 As in American Psychiatric Association (2013)  *Diagnostic and statistical manual of mental disorders, fifth edition*.
